# The association of funding source on effect size in randomized controlled trials: 2013–2015 – a cross-sectional survey and meta-analysis

**DOI:** 10.1186/s13063-017-1872-0

**Published:** 2017-03-14

**Authors:** Alberto Falk Delgado, Anna Falk Delgado

**Affiliations:** 1 Department of Surgical Sciences, Uppsala University Hospital, 75185 Uppsala, Sweden; 2 Department of Clinical Neuroscience, Karolinska Institutet, Stockholm, Sweden; 3 Uppsala University Hospital, Akademiska sjukhuset, 75185 Uppsala, Sweden; 4 Neuroradiology, Karolinska University Hospital, Stockholm, Sweden

**Keywords:** For-profit, Surrogate, Funding, Endpoint, Odds ratio

## Abstract

**Background:**

Trials financed by for-profit organizations have been associated with favorable outcomes of new treatments, although the effect size of funding source impact on outcome is unknown. The aim of this study was to estimate the effect size for a favorable outcome in randomized controlled trials (RCTs), stratified by funding source, that have been published in general medical journals.

**Methods:**

Parallel-group RCTs published in *The Lancet*, *New England Journal of Medicine*, and *JAMA* between 2013 and 2015 were identified. RCTs with binary primary endpoints were included. The primary outcome was the OR of patients’ having a favorable outcome in the intervention group compared with the control group. The OR of a favorable outcome in each trial was calculated by the number of positive events that occurred in the intervention and control groups. A meta-analytic technique with random effects model was used to calculate summary OR. Data were stratified by funding source as for-profit, mixed, and nonprofit. Prespecified sensitivity, subgroup, and metaregression analyses were performed.

**Results:**

Five hundred nine trials were included. The OR for a favorable outcome in for-profit-funded RCTs was 1.92 (95% CI 1.72–2.14), which was higher than mixed source-funded RCTs (OR 1.34, 95% CI 1.25–1.43) and nonprofit-funded RCTs (OR 1.32, 95% CI 1.26–1.39). The OR for a favorable outcome was higher for both clinical and surrogate endpoints in for-profit-funded trials than in RCTs with other funding sources. Excluding drug trials lowered the OR for a favorable outcome in for-profit-funded RCTs. The OR for a favorable surrogate outcome in drug trials was higher in for-profit-funded trials than in nonprofit-funded trials.

**Conclusions:**

For-profit-funded RCTs have a higher OR for a favorable outcome than nonprofit- and mixed source-funded RCTs. This difference is associated mainly with the use of surrogate endpoints in for-profit-financed drug trials.

**Electronic supplementary material:**

The online version of this article (doi:10.1186/s13063-017-1872-0) contains supplementary material, which is available to authorized users.

## Background

Results of well-conducted randomized controlled trials (RCTs) are the most important building blocks of evidence-based medicine and guide clinical management. Clinical decisions based on RCTs rest on the assumption that selection bias is reduced compared with in nonrandomized trials. Randomization improves the likelihood of balance between known and unknown prognostic factors.

However, no study completely lacks confounding factors and biases. Bias can be introduced into RCTs during the entire study course. Imperfect study design, lack of blinding, ascertainment bias, funding, publication bias, and many more factors could all play an important role in influencing the results of RCTs. If there are unreported biases in RCTs influencing clinical guidelines, these guidelines could potentially have been designed on the basis of inadequate medical evidence and could potentially hamper adequate clinical decision making.

Industry sponsorship has been shown to favor the company’s own product [1–3]. Earlier research on industry funding and its impact on outcome in RCTs has been focused on particular fields in medicine. Further, the definition of a favorable outcome varies between different studies or has been defined through the use of scoring systems of trial conclusions [4–6] or on the basis of reported outcomes [3], possibly in itself introducing bias.

The actual treatment effect associated with funding source is unknown. Furthermore, the mechanisms related to more favorable outcome in studies funded by for-profit sources are conflicting. It has been suggested by one group of authors to be caused by the use of surrogate endpoints [7], but other authors have not been able to confirm this [8].

To address the issue of the association of funding source with outcome and follow-up regarding the implementation of the reporting of clinical trials, we evaluated all parallel-group RCTs published in *JAMA*, *The Lancet*, and *New England Journal of Medicine* between 2013 and 2015. The primary aim of this study was to calculate the OR for a favorable outcome in the intervention group compared with the control group in RCTs, stratified by funding source of trials published in 2013–2015, using a meta-analytic approach.

## Methods

### Data source

A librarian performed an electronic search to identify studies published in *JAMA*, *The Lancet*, and *New England Journal of Medicine* between 2013 and 23 September 2015. Trials were identified by using the search terms “randomized controlled trials,” “*N Eng J Med*,” “*Lancet*,” and “*JAMA*” in the PubMed database. A full search strategy is presented in Additional file 1: Table S1. The study followed the Preferred Reporting Items for Systematic Reviews and Meta-Analyses (PRISMA) guidelines.

### Study selection

The following inclusion criteria were used:

1. Only two-armed RCTs were included.
2. RCTs with binary primary endpoints were included.

We decided a priori to study the primary outcome of each study as stated in the published articles. When primary outcome was a continuous variable and the secondary outcome was binary (e.g., survival), the secondary outcome was chosen for evaluation.

The following exclusion criteria were used:

1. Multiarm RCTs
2. Editorials, abstracts, observational studies, and commentaries
3. Non-RCTs

### Data extraction

The primary specified outcome was the OR of a favorable outcome in the intervention group compared with the control group. The intervention group was defined as the group receiving a new treatment compared with a standard treatment or a control group. Favorable outcome was defined as events that were favorable for the patient (e.g., survival, progression free-survival, modified Rankin Scale score 0–2). Unfavorable outcome was defined as events that were unfavorable to the patient (e.g., mortality, myocardial infarction, stroke, modified Rankin Scale score 3–6). OR was calculated as the number of events that occurred in the intervention and control groups. Time-to-survival studies were included, and the number of events that occurred during the study period was used to calculate the OR. If the primary endpoint was unfavorable (e.g., mortality), the number of unfavorable events was subtracted from the total number of patients in the respective treatment arm in order to obtain the number of corresponding favorable (in this case, survival) events to calculate the OR for a favorable outcome.

A clinical endpoint was defined as events with a direct consequence for the patient, such as mortality, stroke, or improvement of symptoms. A surrogate endpoint was defined as a measure with an indirect/unclear consequence for the patient (e.g., biomarkers, imaging, laboratory tests).

One author screened all search results and included studies meeting all inclusion criteria. Outcome data were extracted according to a prespecified protocol for each study by one author. Outcome data were then hidden, and data on funding source were extracted. This procedure was repeated twice. Data on funding were stratified into three groups depending on funding type: for-profit organization (as the only funding source), nonprofit organization (as the only funding source), or mixed funding with both for-profit and nonprofit funding (at any percentage each). Free provision of drugs/devices to studies was defined as funding and thus was assigned to the for-profit-financed trial group if no nonprofit organization participated in the study.

### Statistical analysis

The Open Meta-Analyst (Center for Evidence Synthesis in Health, Brown University, Providence, RI, USA [9]) was used for all analyses. For each study, data were analyzed to estimate the OR for a favorable outcome by the published per-protocol analyses as reported in the published article and according to the intention-to-treat (ITT) population. In the ITT analysis, a single imputation method was used, applying a worst-case scenario with unfavorable outcome imputed for missing data. We obtained summary ORs with corresponding 95% CIs for all outcome analyses. An a priori use of the Mantel-Haenszel method with a random effects model was applied. Outcome heterogeneity was evaluated with Cochrane’s Q test (significance level cutoff value <0.10) and the *I* ^2^ statistic (significance cutoff value >50%). To also include zero total events trials in the meta-analyses, an adjusted continuity correction of 0.5 was applied [10]. To calculate differences between ORs stratified for funding source, a *z*-score was calculated by the logarithm (the summary OR/SE to logarithmic OR), and a two-tailed *z*-test was calculated to obtain a *p* value for the difference between ORs. To explore factors associated with the magnitude of OR, we performed a metaregression where the dependent variable was OR for a favorable outcome and the explanatory variables were funding source, population size, intervention type, journal, and type of endpoint (clinical/surrogate). A logistic mixed effects model for the metaregression implemented in R [11] as a plugin for Open Meta-Analyst was used because it allows for both within-study variation and between-study variation. To investigate differences in the OR between different subgroups, prespecified sensitivity analyzes were performed, including medical fields, type of intervention, type of endpoint, blinding, and comparator (placebo/nonplacebo) in drug trials. Because we had a rather strict definition of funding source in which free provision of drug was considered as sponsorship by industry, we carried out a sensitivity analysis excluding studies with industry-funded free provision of drugs.

## Results

### Description of included studies

The electronic search identified a total of 1149 publications, of which 509 RCTs fulfilled all inclusion criteria. Topics covered all medical disciplines. One hundred forty-eight (29%) trials were exclusively funded by for-profit organizations, 117 (23%) trials were jointly funded by for-profit and nonprofit organizations, and 244 (48%) trials were exclusively financed by nonprofit organizations.

Characteristics of the included studies are presented in Table 1. There were no differences in study sample size or the use of clinical and/or surrogate endpoints between funding sources (Table 1).

**Table 1:** Characteristics of 509 included studies

	For-profit (*n* = 148)	Mixed profit and nonprofit (*n* = 117)	Nonprofit (*n* = 244)
Medical journal, *n* (%)			
*JAMA*	17 (11%)	37 (32%)^a^	63 (26%)^a^
*The Lancet*	45 (30%)	33 (28%)	91 (37%)
*New England Journal of Medicine*	86 (58%)	47 (40%)^a^	90 (37%)^a^
Sample size			
Median	800	637	647
Intervention type, *n* (%)			
Drug	118 (80%)	65 (56%)^a^	93 (38%)^a,b^
Device	18 (12%)	6 (5%)	16 (7%)
Procedure	4 (3%)	7 (6%)	42 (17%)^a,b^
Procedure and/or device/drug/behavior	5 (3%)	21 (18%)^a^	21 (9%)^b^
Behavior	0 (0%)	1 (1%)	45 (18%)^a,b^
Other	3 (2%)	17 (15%)^a^	27 (11%)^a^
Endpoint, *n* (%)			
Clinical	110 (74%)	91 (78%)	196 (80%)
Surrogate	38 (26%)	26 (22%)	48 (20%)

^a^Denotes statistical significant difference compared with the for-profit-funded group (*p* < 0.05)

^b^Denotes statistical significant difference compared with mixed group funded research (*p* < 0.05)

### Association of funding source on outcome

Calculation of the effect size for a favorable outcome in the intervention group compared with the control group was performed for all included studies and stratified for funding source. The OR for a favorable outcome in for-profit funded RCTs was 1.92 (95% CI 1.72–2.14), which was higher than in the mixed-funded RCTs (OR 1.34, 95% CI 1.25–1.43). For-profit-funded RCTs had a higher OR for a favorable clinical outcome than nonprofit-funded RCTs (OR 1.32, 95% CI 1.26–1.39). No statistical differences were found between the per-protocol and ITT analyses (Table 2). There was considerable heterogeneity between the included studies (*I* ^*2*^ 73% to 96%).

**Table 2:** Meta-analysis comparing OR of favorable outcome, stratified by result analysis, primary outcome, and funding source

Funding source	Per-protocol, OR (95% CI)	Intention-to-treat, OR (95% CI)	Clinical outcome, OR (95% CI)	Surrogate outcome, OR (95% CI)
For-profit	1.92 (1.72–2.14)	1.74 (1.58–1.92)	1.50 (1.40–1.61)	3.72 (2.53–5.51)
Mixed	1.34 (1.25–1.43)^a^	1.30 (1.22–1.38)^a^	1.27 (1.16–1.34)^a^	1.57 (1.26–1.95)^a^
Nonprofit	1.32 (1.26–1.39)^a^	1.33 (1.26–1.40)^a^	1.27 (1.19,1.37)^a^	1.62 (1.46–1.76)^a^

^a^Statistical difference compared with for-profit-funded research (*p* < 0.05)

### Subgroup analyses, metaregression, and sensitivity analyses

#### Medical field

Effect estimates in different medical fields and different funding sources are presented in Table 3. For-profit-funded RCTs had a higher OR for a favorable outcome than nonprofit-funded RCTs for all medical fields except diabetes, cardiovascular, and autoimmunity. The OR was higher in for-profit-funded research than for mixed-funded research in cardiovascular and autoimmunity studies. Almost exclusively, behavioral intervention trials were nonprofit-funded (*n* = 44). Excluding behavioral intervention trials did not change the overall results for the nonprofit group (OR 1.31, 95% CI 1.23–1.41). There were no differences in the ORs between different funding sources in device and procedure trials.

**Table 3:** Subgroup analysis of medical fields, stratified by funding source, comparing OR of favorable primary outcome between intervention and control groups

Funding source	Cancer, OR (95% CI)	Cardiovascular, OR (95% CI)	Infectious disease, OR (95% CI)	Neurology, OR (95% CI)	Autoimmunity, OR (95% CI)	Diabetes, OR (95% CI)	Other, OR (95% CI)
Profit	1.89 (1.65–2.17)	1.83 (1.50–2.22)	1.96 (1.57–2.44)	2.12 (1.36–3.30)	4.34 (2.72–6.94)	1.86 (1.00–3.45)	2.52 (1.59–3.97)
Mixed funding	1.55 (1.26–1.90)	1.17 (1.03–1.20)^a^	1.45 (1.17–1.79)	1.57 (1.16–2.14)	1.46 (1.05–2.03)^a^	1.05 (0.91–1.20)	1.49 (0.96–2.07)
Nonprofit	1.13 (0.87–1.43)^a^	1.56 (1.37–1.76)^b^	1.33 (1.18–1.50)^a^	1.13 (0.97–1.32)^a^	2.39 (1.20–4.77)	1.12 (0.96–1.29)	1.31 (1.23–1.41)^a^

^a^Denotes statistical difference compared with the for-profit funded studies (*p* < 0.05)

^b^Denotes statistical difference compared with mixed funded studies (*p* < 0.05)

### Metaregression

Metaregression was performed to assess the role of moderator variables on the effect size. The following factors were included: sample size, medical journal, clinical/surrogate endpoint, intervention type, and funding source. The metaregression analysis showed that differences in OR could be explained by reporting on surrogate compared with clinical endpoints, as well as by funding source, such as for-profit funding compared with other funding sources. Furthermore, drug trials are associated with a higher OR than device trials and procedure trials in combination with other types of intervention. Smaller sample size and medical journal were not related to differences in effect size (Table 4).

**Table 4:** Multivariate metaregression examining factors associated with effect size

Factor	Exponentiated coefficient, OR (95% CI)	*p* Value
Medical journal		
*The Lancet*	Reference	
*JAMA*	−0.21 (−0.17, 0.12)	0.77
*New England Journal of Medicine*	0.024 (−0.10, 0.15)	0.70
Sample size		
Mean	0 (0, 0)	0.76
Intervention type		
Drug	Reference	
Device	−0.21 (−0.40, −0.01)	0.04
Procedure	0.04 (−0.15, 0.23)	0.67
Procedure and/or device/drug	−0.20 (−0.40, −0.00)	0.049
Behavior	0.02 (−0.19, 0.23)	0.85
Other	−0.18 (−0.35, 0.00)	0.053
Endpoint		
Clinical	Reference	
Surrogate	0.38 (0.25, 0.51)	<0.001
Funding source		
Profit	Reference	
Mixed	−0.22 (−0.37, 0.07)	0.005
Nonprofit	−0.28 (−0.42, −0.15)	<0.001

### Clinical and surrogate endpoints

The OR of a favorable outcome for clinical endpoints was significantly higher in for-profit funded RCTs (OR 1.50, 95% CI 1.40–1.61) than in mixed-funded RCTs (OR 1.27, 95% CI 1.16–1.34) and nonprofit-funded RCTs (OR 1.27, 95% CI 1.19–1.37). This difference in OR between for-profit and nonprofit/mixed-funded RCTs was also seen in surrogate endpoints (Table 2).

When we stratified drug trials into clinical and surrogate endpoints, we found no difference in the OR of clinical endpoints between funding sources. However, we found significantly higher OR for surrogate endpoints in for-profit-funded drug trials (OR 4.10, 95% CI 2.64–6.37) than in nonprofit-funded drug trials (OR 1.66, 95% CI 1.11–2.47) (Table 5).

**Table 5:** Subgroup analyses comparing OR of favorable outcome, stratified by intervention type and funding source

Funding Source	Drug trials, OR (95% CI)	No.	Excluding drug trials, OR (95% CI)	No.	Drug trial, clinical endpoints, OR (95% CI)	No.	Drug trial, surrogate endpoints, OR (95% CI)	No.	Drug trial, excluding trials with free provision of study drug, OR (95% CI)	No.	Drug trial, excluding trials with free provision of study drug, OR (95% CI)	No.	Nonplacebo drug trials, OR (95% CI)	No.	Double-blind drug trials, OR (95% CI)	No.	Non-double-blind drug trials, OR (95% CI)	No.
For-profit	2.41 (2.05–2.83)	118	1.44 (1.23–1.68)	30	1.55 (1.43–1.67)	86	4.1 (2.64–6.37)	31	2.41 (2.05–2.83)	118	2.29 (1.96–2.70)	73	1.83 (1.45–2.31)	45	2.01 (1.78–2.29)	88	2.12 (1.48–3.03)	30
Mixed	1.63 (1.46–1.84)^a^	65	1.37 (1.05–1.79)	52	1.37 (1.23–1.51)	50	2.01 (1.46–2.77)	15	1.62 (1.42–1.84)	43	1.47 (1.30–1.67)^a^	34	1.50 (0.96–1.39)^a^	31	1.56 (1.35–1.81)	31	1.26 (1.11–1.42)^a^	34
Nonprofit	1.36 (1.24–1.50)^a^	93	1.38 (1.28–1.48)	151	1.29 (1.18–1.43)	82	1.66 (1.11–2.47)^a^	11	1.36 (1.24–1.50)^a^	93	1.41 (1.25–1,57)^a^	44	1.37 (1.11–1.71)	49	1.41 (1.21–1.63)^a^	48	1.25 (1.10–1.42)^a^	45

^a^Denotes statistical difference compared with profit funded studies (*p* < 0.05)

^b^Denotes statistical difference compared with mixed funded studies (*p* < 0.05)

### Drug trials

Excluding drug trials changed the OR from a statistical difference depending on funding source to a nonsignificant difference in OR between funding. Placebo-controlled for-profit drug trials had a higher OR than mixed- and nonprofit-funded placebo drug trials. Double-blind drug trials and non-double-blind trials had a higher OR in for-profit-financed trials than nonprofit-financed trials (Table 5). Non-placebo-controlled drug trials with for-profit funding had a higher OR than mixed-funded trials, but there was no difference from nonprofit-funded research.

## Discussion

In this study, we calculated the OR for a favorable outcome in 509 RCTs published in general medical journals between 2013 and 2015, stratified by funding source, and we investigated the underlying mechanisms for differences in OR. RCTs funded by for-profit organizations had a higher OR for a favorable outcome than both nonprofit-funded and mixed-funded RCTs. The OR for a favorable outcome was higher for both clinical and surrogate endpoints in for-profit-funded trials than for RCTs with other funding. The significant difference in OR between for-profit- and nonprofit-funded RCTs was lost when we excluded drug trials, indicating that a higher OR is associated with drug trials. After stratifying drug trials into clinical endpoints, we found no differences in OR between funding sources. For-profit-financed drug trials in which researchers reported on surrogate endpoints had a significantly higher OR of a favorable outcome than nonprofit-financed drug trials.

We speculated that the resulting OR stratified for funding source would differ between the per-protocol and ITT analyses owing to differences in missing data between studies. However, consistent with earlier observations, our results contradicted this speculation, showing no difference between the ITT and per-protocol analyses [12].

The strengths of this study are the large number of included studies (*n* = 509) from recent years. Trials from all medical fields were included to fully study the concept of funding influencing trial results. We analyzed studies with binary outcomes, which are more commonly associated with hard clinical endpoints such as progression-free survival than continuous variables, which are more often surrogate markers such as blood pressure [13]. In addition, we included studies with time-to-survival rates and recorded the actual events at a certain time point, because those studies are often conducted in cancer fields and often financed by industry. Using a meta-analytic technique, we were able to quantify the effect size of a favorable outcome in the intervention group compared with the control group on the basis of actual study event rates of outcomes rather than scoring systems. To our knowledge, the aggregated effect size based on funding source has not been previously reported. To limit bias in selecting reported outcomes we prespecified that we would study the primary study outcome as reported in the published articles.

Limitations of this study are due mainly to the statistical constraints of a meta-analysis combining different study designs, populations, and interventions. A high heterogeneity was expected in this study because different studies with completely different outcomes from all medical fields were included, along with a variety of types of interventions. Also, the study design varied between trials. Altogether, this yielded a high heterogeneity. However, in order to comprehensively explore the size and magnitude of the association between funding source and study outcome, a meta-analysis with appropriate sensitivity analyses was warranted.

There are several approaches to addressing heterogeneity [14]. We chose to explore heterogeneity by applying metaregression analysis and a random effects model. The metaregression analysis revealed that the use of surrogate endpoints in clinical trials was associated with higher OR than the use of clinical endpoints. Also, drug trials were associated with a higher OR than device trials or procedure trials in combination with other types of interventions. Furthermore, the metaregression analysis showed that for-profit-funded trials were associated with higher OR than other funding sources. The inclusion of small studies and medical journal type in the model did not have a moderating variable effect on the meta-analysis effect size (OR). These results contradict the hypothesis that smaller studies are related to higher treatment effects or that higher treatment effect is associated with publication (e.g., *New England Journal of Medicine* compared with *The Lancet* or *JAMA*). Given the diversity of clinical conditions and outcomes that were pooled in our analysis, we tried to adjust for differences in baseline characteristics by performing relevant sensitivity analyses.

Our findings are in line with earlier studies demonstrating an association between for-profit-funded research and a more favorable outcome [1, 3, 5, 15]; however, these studies were based on data published 10–15 years ago. Previous research has been focused on specific medical fields, such as cardiovascular research [3], nutrition [16], or drug trials [5]. Our stratified analysis for different medical fields showed a higher OR for a favorable outcome in all groups except autoimmunity, cardiovascular, and diabetes, with the highest absolute OR being in autoimmunity trials. Our study shows that the association between a higher OR for a favorable outcome and for-profit-financed trials is consistent in all medical fields. The OR for a favorable outcome in for-profit-financed drug trials was higher in placebo-controlled studies and double-blind studies than in nonprofit-financed drug trials. Free provision of drugs by industry is common in RCTs performed today, here defined as mixed funding if the trial was otherwise funded by a nonprofit organization. Excluding these trials did not change the OR.

Previous studies of the association between funding source and study outcome or conclusion have defined a favorable outcome on the basis of either statistical significance [16] or a scoring system [3, 5]. The ORs in for-profit-funded research in these studies have not been statistically different from nonprofit-funded studies [5, 17, 18]. Two exceptions are trials investigating nicotine replacement therapy [19] and glucosamine [20]. The authors of a Cochrane report published in 2012 stated that the higher OR in industry-sponsored studies could be mediated by unknown factors regarded as “metabias” [1].

There are several hypotheses explaining the differences in rates of favorable outcome between studies. Differences due to variations in methodological quality are unlikely in studies published in the included high-impact journals, because study quality in general is higher in high-impact journals than in other medical journals [21]. Probably, this is because many drug trials are almost consistently conducted by contract research organizations, which are frequently multi-billion-dollar organizations that have more experience in conducting trials than many academic centers and also have more risk safeguards in place than many academic or nonprofit centers. This has been further evaluated in a study by Boutron et al. [22], who reported on the presence of “spin” in RCTs with nonsignificant results for the primary endpoint. In the study by Boutron et al., spin was reported in studies published mainly in lower-impact journals. Previous studies do not support the notion that industry funding is associated with inferior study quality [4, 23, 24]. Our results show that study blinding and comparator group (e.g., active control or placebo) poorly explain differences in effect size between funding sources. Our findings suggest that the use and selection of surrogate markers as primary endpoints partly explain the difference in effect size between funding sources.

One could argue that the differences in outcome between funding types can be explained by publication bias whereby industry groups rarely publish negative results and drug industry-funded research is less likely to be published [15]. In accordance with these concepts, research funded by drug companies has been reported to be subject to selective publication, selective reporting, and multiple publications [25]. Previous researchers reported that pharmaceutical companies have suppressed studies reporting severe adverse effects [26, 27]. Kasenda et al. [28] reported on reasons for discontinuation of clinical trials. For-profit-funded studies were less likely than nonprofit-funded trials to be discontinued because of poor recruitment. Do pharmaceutical industries have a better availability of effective drugs? This is partly contradicted by a study from Kasenda et al., who found discontinuation of clinical trials because of futility in equal proportions between for-profit- and nonprofit-funded trials [28]. In analyzing drug trials, they found that there were no differences in effect sizes for clinical endpoints. This contradicts the hypothesis that for-profit organizations have more effective drugs. In addition, as reviewed by Svensson et al., several drugs approved on the basis of surrogate endpoints have later been shown to have substantial side effects, impacting patients’ clinical course negatively [29].

In a RCT, Kesselheim et al. [30] compared how physicians interpret trials depending on methodological differences and funding sources. For-profit funding reduced physicians’ willingness to believe the data. In the last decade, several measures have been taken to reduce selective reporting in trials through the introduction of trial registration [31] and the Consolidated Standards of Reporting Trials (CONSORT) guidelines [32]. The existence of industry-financed RCTs is essential for the development of new drugs and modern treatments. However, physicians’ mistrust of for-profit-funded research is of great concern. Despite the latest year’s implementation of trial registration and methodological guidelines for RCTs, the present study shows a clear association between for-profit-financed studies and a higher OR for a favorable outcome than for RCTs supported by other types of funding.

The present study shows evidence that for-profit-financed RCT researchers still report higher ORs, despite recent years’ efforts to increase good research conduct and transparency in RCTs. Recently, the International Committee of Medical Journal Editors proposed the concept of data sharing for clinical trials, enabling secondary analyses of studies [33]. In a previous study, Ebrahim et al. [34] evaluated reanalyses of published RCTs and found that 35% of the reanalyses could alter conclusions from those originally published, were the original authors to have concluded that they were inept in their analysis, further indicating this need for an open access to published study data. Other strategies can contain greater public funding of RCTs or “money-blind” independent organizations that would help to develop trial methodology on the basis of industry funding. The findings of the present study indicate that the use of surrogate markers as primary endpoints should be clearly stated as such in the abstracts of the trials, in order not to overestimate the potential clinical benefit for the individual patients.

## Conclusions

Our study shows evidence supporting the hypothesis that for-profit-financed RCTs are associated with a higher OR for a favorable outcome of a new treatment than nonprofit- and mixed-funded RCTs, and that this difference is associated mainly with the use of surrogate endpoints in for-profit-financed drug trials. However, for-profit-financed drug trials with clinical endpoints have ORs equal to those of trials with other funding sources.

## Additional file

Additional file 1: Supplementary material. (DOCX 14 kb)

## Abbreviations

CONSORT: Consolidated Standards of Reporting Trials
ITT: Intention to treat
PRISMA: Preferred Reporting Items for Systematic Reviews and Meta-Analyses
RCT: Randomized controlled trial
