# Supplementary material for: The association of funding source on effect size in randomized controlled trials: 2013–2015 – a cross-sectional survey and meta-analysis
Source: Trials. 2017 Mar 14;18:125. doi: 10.1186/s13063-017-1872-0 (PMC5351064; doi:10.1186/s13063-017-1872-0)
Supplement: Supplementary file 1 — Supplementary material. (DOCX 14 kb) [file 13063_2017_1872_MOESM1_ESM.docx]

**Additional file 1: Table S1.**

| Search,Query,Items found,Time |  |  |  |  |
| --- | --- | --- | --- | --- |
| #8,"Search (((((""JAMA""[Journal]) OR ""Lancet""[Journal]) OR ""N Engl J Med""[Journal] (randomized OR randomised)) AND ( ""2013/01/01""[PDat] : ""2015/12/31""[PDat] ))) OR ((((""JAMA""[Journal]) OR ""Lancet""[Journal]) OR ""N Engl J Med""[Journal]) AND Randomized Controlled Trial[ptyp] AND ( ""2013/01/01""[PDat] : ""2015/12/31""[PDat] )) Filters: Publication date from 2013/01/01 to 2015/12/31",1149,07:30:28 | | | | |
| #7,"Search ((""JAMA""[Journal]) OR ""Lancet""[Journal]) OR ""N Engl J Med""[Journal] (randomized OR randomised) Filters: Publication date from 2013/01/01 to 2015/12/31",1148,07:29:16 | | | | |
| #5,"Search ((""JAMA""[Journal]) OR ""Lancet""[Journal]) OR ""N Engl J Med""[Journal] Filters: Publication date from 2013/01/01 to 2015/12/31",12422,07:28:36 | | | | |
| #6,"Search ((""JAMA""[Journal]) OR ""Lancet""[Journal]) OR ""N Engl J Med""[Journal] Filters: Randomized Controlled Trial. Publication date from 2013/01/01 to 2015/12/31",809,07:28:12. | | | | |
| #4,"Search ((""JAMA""[Journal]) OR ""Lancet""[Journal]) OR ""N Engl J Med""[Journal]",269460,07:27:38 | | | | |
